# Effect of sodium glucose cotransporter 2 inhibitors on cardiac function and cardiovascular outcome: a systematic review

**DOI:** 10.1186/s12947-019-0177-8

**Published:** 2019-11-13

**Authors:** Koichiro Matsumura, Tetsuro Sugiura

**Affiliations:** Department of Cardiology, Kansai Medical University Medical Center, 10-15, Fumizono-cho, Moriguchi, Osaka 570-8507 Japan

**Keywords:** Diabetes mellitus, Cardiac function, SGLT2 inhibitor

## Abstract

A high incidence of left ventricular diastolic dysfunction and increased risk of cardiovascular events have been reported in patients with diabetes mellitus. Sodium glucose cotransporter 2 (SGLT2) inhibitors selectively inhibit kidney glucose and sodium reabsorption, and cardiovascular benefits of SGLT2 inhibitors beyond other antidiabetic drugs have been reported in type 2 diabetes mellitus (T2DM) clinical trials. However, underlying mechanisms contributing to the improvement of cardiovascular outcomes have not been clearly identified. In this review, likely mechanisms of SGLT2 inhibitors contributing to a favorable cardiovascular outcomes are discussed based on experimental and clinical studies on cardiac function.

## Background

Diabetes mellitus is associated with increased risk of cardiovascular events including cardiovascular death and hospitalization from heart failure. Diabetic cardiomyopathy affects cardiac function as well as cardiac structure such as left ventricular (LV) hypertrophy and fibrosis, which are considered as major contributors of cardiovascular events [1–3]. Sodium glucose cotransporter 2 (SGLT2) inhibitors have newly emerged as an anti-hyperglycemic drug for type 2 diabetes mellitus (T2DM) by inhibiting glucose and sodium reabsorption in the kidney. In large clinical trials (EMPA-REG OUTCOME, CANVAS Program and DECLARE-TIMI 58), SGLT2 inhibitors have shown to improve long-term clinical outcome including all cause mortality, cardiovascular death and heart failure hospitalization in T2DM [4–6]. Meta-analysis also showed clinical benefit of SGLT2 inhibitors in reducing risk of myocardial infarction, stroke and cardiovascular death in patients with established atherosclerotic cardiovascular disease [7]. Moreover, SGLT2 inhibitors showed risk reduction of heart failure hospitalization in T2DM patients, suggesting that SGLT2 inhibitors play a key role in the improvement of cardiac function in diabetic cardiomyopathy [8]. More recently, DAPA-HF reported that dapagliflozin improved cardiovascular outcomes among patients with heart failure with reduced ejection fraction regardless of diabetic status, therefore SGLT2 inhibitors have been expected that its pharmacological action is beyond antidiabetic drug [9]. To elucidate the effect of SGLT2 inhibitors on cardiovascular event reduction, it is important to clarify the mechanisms contributing to the cardioprotective effect of SGLT2 inhibitors. Accordingly, we reviewed the effect of SGLT2 inhibitors on cardiac function in animal models and clinical studies, and discussed the underlying mechanisms contributing to cardioprotection.

## Review methods

We searched English language literatures using PubMed. Search terms were “sodium glucose cotransporter 2”, “cardiac function” and “left ventricular”. In addition, term of “empagliflozin”, “canagliflozin” or “dapagliflozin” was searched in PubMed and the articles evaluating cardiac function were extracted. Article relevance was assessed by subject and study design.

### Effect of SGLT2 inhibitors on cardiac function

In experimental diabetic cardiomyopathy models, SGLT2 inhibitors improved both cardiac systolic and diastolic function (Table 1). Moreover, LV pressure-volume loop analysis in vivo showed improvement of end-systolic and end-diastolic pressure volume relationships by SGLT2 inhibitors [10–14]. Pathological experimental studies showed that SGLT2 inhibitors attenuated LV fibrotic area [11, 12, 15–17]. These experimental data indicate that plasma volume reduction by SGLT2 inhibitors strongly contributed to the attenuation of pressure-overload-induced cardiac fibrosis and remodeling [18].

**Table 1:** Effect of SGLT2 inhibitors on cardiac function

	Subjects	Observation period	Improved cardiac parameters	Other evaluation^a^	SGLT2 inhibitor	Reference
Clinical studies	T2DM	12 weeks	LV mass index, e’	No	Empagliflozin	[23]
	T2DM	12 weeks	LV mass index, E’	No	Canagliflozin	[24]
	T2DM	24 weeks	LV mass index, LA volume index, E/e’	No	Dapagliflozin	[25]
	T2DM	24 weeks	EF, E/E’	No	Tofogliflozin	[26]
	T2DM	24 weeks	EDV	CMR	Empagliflozin	[27]
Animal experiments	db/db mice	5 weeks	E’/A’, E/E’, CO, SV, LA	No	Empagliflozin	[31]
	ob/ob mice	6 weeks	E, DT, Tau, EDPVR	PV analysis	Empagliflozin	[10]
	SKO mice	8 weeks	EF, E/A, DT, IVRT, LV wall thickness	CMR	Dapagliflozin	[42]
	BTBR mice	8 weeks	EF, FS, EDV, ESV, LV wall thickness	No	Dapagliflozin	[15]
	CRDH rats	11 weeks	LV mass, ESd, E/A, DT, IVRT	No	Empagliflozin	[32]
	db/db mice	4 weeks	E	No	Empagliflozin	[22]
	SHR rats	12 weeks	EDV, ESV, ESPVR, dP/dt	PV analysis	Empagliflozin	[11]
	KK-Ay mice	8 weeks	EF, FS, EDd, E/A, LV wall thickness	No	Empagliflozin	[16]
	Human, mice and ZDF rats	30 min	E/A, IVRT	No	Empagliflozin	[43]
	Pre-DM rats	4 weeks	EF, ESd, LV wall thickness	PV analysis	Dapagliflozin	[12]
	Non-DM mice	2 weeks	EF, CO	Ex vivo perfused hearts model	Empagliflozin	[19]
	Non-DM pigs	8 weeks	EF, LV mass, EDV, ESV, GLS, GCS, GRS	CMR	Empagliflozin	[20]
	Non-DM rats	10 weeks	EF, LV mass	No	Empagliflozin	[21]
	Non-DM rats	145 min	PRSW	PV analysis	Canagliflozin	[13]
	Non-DM rats	4 weeks	LV mass, ESd, Tau, Wall stress	PV analysis	Empagliflozin	[14]
	Non-DM mice	4 weeks	EF, FS, ESd, LV mass	No	Dapagliflozin	[44]
	DCM mice	6 weeks	EF, EDd, ESd	No	Empagliflozin	[17]

A velocity of late mitral flow, A’ late peak velocity of septal annulus, *CMR* Cardiac magnetic resonance, *CO* Cardiac output, *CRDH* Cohen-Rosenthal diabetic hypertensive, *DCM* Dilated cardiomyopathy, DT E wave deceleration time, E velocity of early mitral flow, e’ early peak velocity of lateral annulus, E’ early peak velocity of septal annulus, *EDd* End diastolic diameter, *EDPVR* End diastolic pressure volume relationship, *EDV* End diastolic volume, *EF* Ejection fraction, *ESd* End systolic diameter, *ESPVR* End systolic pressure volume relationship, *ESV* end systolic volume, *FS* Fractional shorting, *GCS* Global circumferential straining, *GLS* Global longitudinal strain, *GRS* Global radial strain, *IVRT* Isovolumetric relaxation time, *LA* Left atrial, *LV* Left ventricular, *PRSW* Preload recruitable stroke work, *PV* Pressure-volume, *SGLT2* Sodium glucose cotransporter 2, *SHR* Spontaneous hypertensive rats, *SKO* Seipin knockout, *SV* Stroke volume, *T2DM* Type 2 diabetes mellitus, *ZDF* Zucker diabetic fatty

^a^Other cardiac functional evaluation except echocardiography

In models of myocardial ischemia, SGLT2 inhibitors not only suppressed exacerbation of systolic and diastolic cardiac dysfunction but also prevented LV remodeling and expansion of fibrosis area following ischemic myocardial injury [12, 13, 19–21]. These authors suggested that SGLT2 inhibitors reduced mitochondrial damage by stimulating mitochondrial biogenesis, which resulted in the normalization of myocardial uptake and oxidation of glucose and fatty acids. Furthermore, SGLT2 inhibitors increased circulating ketone levels and myocardial ketone utilization indicating enhancement of myocardial energetics [20–22]. Evidenced from these reports, SGLT2 inhibitors also exert cardioprotective effect exposed to ischemia.

Two experimental studies investigated cardiac function of SGLT2 inhibitor alone and combined therapy with SGLT2 inhibitor and DPP4 inhibitor. In a mice model, Ye et al. compared three groups; control, dapagliflozin alone and combined therapy with dapagliflozin and saxagliptin [15]. Both dapagliflozin alone and combined therapy groups showed a significant improvement of LV systolic function, LV end-diastolic and end-systolic volume compared to the control. Moreover, combined therapy group showed a larger improvement of LV end-diastolic and end-systolic volume compared to dapagliflozin alone group. Tanajak et al. compared cardiac protective effect of dapagliflozin vs. vildagliptin after ischemia-reperfusion injury in pre-diabetic rats, which showed that dapagliflozin had a greater efficacy than vildagliptin in improving LV dysfunction and infarct size [11]. Combined therapy with dapagliflozin and vildagliptin showed the greatest efficacy in attenuating LV dysfunction and infarct size. However, human study is needed to define the clinical significance of combined SGLT2 inhibitor and dipeptidyl peptidase 4 inhibitor therapy.

Several clinical studies have reported the effect of SGLT2 inhibitors on cardiac function in T2DM (Table 1). EMPA-REG OUTCOME trial retrospectively evaluated the effect of empagliflozin on cardiac function [23]. In this analysis, transthoracic echocardiogram was performed before and 3 months after initiation of empagliflozin in 10 patients with T2DM. This was a single arm and small number analysis, but showed that short-term empagliflozin treatment resulted in a significant improvement of diastolic function and reduction of LV mass index in T2DM patients with established cardiovascular disease. Matsutani et al. prospectively evaluated transthoracic echocardiogram at baseline and 3 months after additional treatment with canagliflozin in 37 T2DM patients and showed improvement of LV diastolic function and reduction of LV mass index [24]. Although brain natriuretic peptide level did not change between baseline and at 6 months of dapagliflozin treatment, Soga et al. showed improvement of diastolic function as well as reduction of LV mass index and left atrial volume index in 58 T2DM patients with previous history of heart failure [25]. These clinical reports indicate that SGLT2 inhibitors have a favorable effect on diastolic function and LV mass. However, these reports were single arm evaluation regarding the effect of SGLT2 inhibitor on cardiac function. Recently, we compared tofogliflozin and propensity-matched antidiabetic therapy not taking SGLT2 inhibitor, and found that tofogliflozin showed a significant improvement of systolic and diastolic function compared to the controls [26].

Cohen et al. investigated the effect of empagliflozin on cardiac functional and structural changes in patients with T2DM treated with standard glucose lowering therapy plus empagliflozin using cardiac magnetic resonance compared with control patients. As a results, LV end-diastolic volume reduced significantly after 6 months treatment of empagliflozin compared with control patients despite of no significant difference in LV mass [27]. Authors concluded that beneficial effect of SGLT2 inhibitor was due to functional improvement from reduction of plasma volume rather than structural remodeling.

### Underlying mechanisms of SGLT2 inhibitor and cardiovascular outcomes

**Fig. 1:**
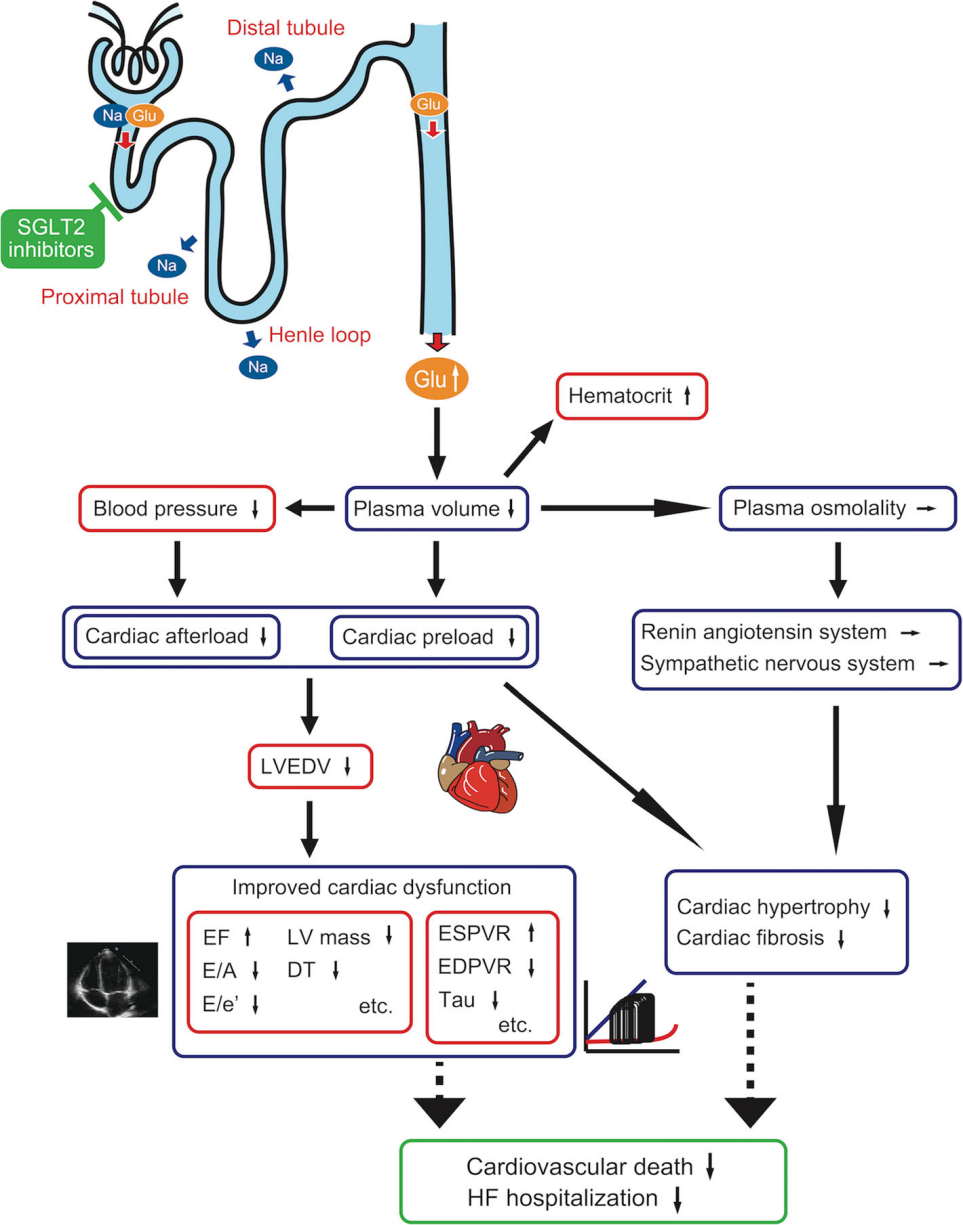
Effect of SGLT2 inhibitors on cardiac function and cardiovascular outcome. Osmotic diuresis mainly caused by urine glucose excretion leads to plasma volume reduction without activating renin angiotensin system and sympathetic nervous system. Plasma volume reduction leads to decreased cardiac workload resulting in the improvement of cardiac function and hence, favorable cardiovascular outcome. Blue box; functional and structural changes, Red box; clinical parameters, Green box; clinical outcome.tab

SGLT2 receptor is located in the proximal tubule of the kidney, where it mediates approximately 90% of renal glucose reabsorption by coupling with sodium reabsorption at 1:1 ratio [28]. Inhibition of SGLT2 receptor leads to increase of urine glucose and sodium excretion, but the increase in urine sodium excretion by SGLT2 inhibitors appears to be transient [29, 30]. This is probably caused by accelerated sodium reabsorption at the proximal tubule, henle loop and distal tubule against inhibition of sodium reabsorption at SGLT2 receptor (Fig. 1). In contrast, continuous urine glucose excretion is demonstrated with SGLT2 inhibitor treatment in many human and experimental studies [30–36]. Therefore, osmotic diuresis observed with SGLT2 inhibitor treatment is mainly caused by urine glucose excretion.

Experimental and clinical studies have shown no activation of renin angiotensin system by SGLT2 inhibitors [29, 37]. Moreover, Matsutani et al. showed that canagliflozin caused no exacerbation of autonomic function as assessed by baroreflex sensitivity and frequency domain analysis of heart rate variability, which suggest that canagliflozin improved LV diastolic function without activating sympathetic nervous system [24]. Blood pressure lowering effect of SGLT2 inhibitor also caused no compensatory increase in heart rate in EMPA-REG OUTCOME trial, indicating that there was no further sympathetic nervous system activation after SGLT2 inhibitor treatment [38, 39]. As evidenced from these studies, diuresis caused by urine glucose excretion results in continuous but mild intravascular fluid reduction without activating renin angiotensin system and sympathetic nervous system [30, 33, 37], because serum glucose has quite small effect on plasma osmolality compared to serum sodium. Therefore, urine glucose excretion, not accompanied by natriuresis, cause not only reduction of cardiac preload but also afterload without activating renin angiotensin system and sympathetic nervous system (Fig. 1).

Increase in hematocrit due to reduction in plasma volume was observed in patients treated with empagliflozin [29]. Moreover, increase of hematocrit after empagliflozin treatment was associated with more than 50% reduction in cardiovascular mortality [40, 41]. These data indicate that decreased circulatory volume by empagliflozin, especially reduction of LV filling pressure, is an important mechanism contributing to a favorable cardiovascular outcome. Thus, improvement of LV function by SGLT2 inhibitors prevented further cardiac morphologic changes and hence, result in favorable cardiovascular outcomes.

DAPA-HF, a large randomized clinical trial, investigated whether dapagliflozin improves long-term cardiovascular outcomes among patients with heart failure with reduced ejection fraction regardless of diabetic status [9]. Dapagliflozin significantly reduced cardiovascular death and heart failure events not only in T2DM patients but also in non-diabetic patients. This study indicates that underlying mechanisms of SGLT2 inhibitors for the improvement of cardiovascular outcomes is independent of glucose lowering effect. Further study using novel cardiac imaging modalities is needed to confirm the relationship between SGLT2inhibitors on cardiac function and a favorable cardiovascular outcome.

## Conclusions

Cardioprotective effect of SGLT2 inhibitors is due to reduction of plasma volume from continuous urine glucose excretion without activating renin angiotensin system and sympathetic nervous system. Therefore, SGLT2 inhibitors have a favorable effect on cardiac function as well as cardiac structure and hence, improvement of cardiovascular outcome.

## Data Availability

The datasets used and/or analyzed during the current study are available from the corresponding author on reasonable request.

## Abbreviations

LV: Left ventricular
SGLT2: Sodium glucose cotransporter 2
T2DM: Type 2 diabetes mellitus
